# Initial results of efficacy and safety of Sofosbuvir among Pakistani Population: A real life trial - Hepatitis Eradication Accuracy Trial of Sofosbuvir (HEATS)

**DOI:** 10.12669/pjms.331.12352

**Published:** 2017

**Authors:** Zahid Azam, Muhammad Shoaib, Masood Javed, Muhammad Adnan Sarwar, Hafeezullah Shaikh, Nasir Khokhar

**Affiliations:** 1 Prof. Zahid Azam, FCPS (Gastro); FCPS (Med); M.Sc (Clinical Research). Professor of Medicine & Gastroenterology, Gastroinstinal and Liver Practice (GILP) and Dow University of Health Sciences, Karachi, Pakistan; 2 Dr. Muhammad Shoaib, FCPS (Gastroenterology). Consultant Gastroenterologist, Memom Medical Institute Hospital, Karachi, Pakistan; 3 Dr. Masood Javed, FCPS, Associate Professor, Punjab Medical Collage Faisalabad, Pakistan; 4 Dr. Muhammad Adnan Sarwar, FCPS(Medicine), Senior Registrar DHQ Hospital Faisalabad, Pakistan; 5 Dr. Hafeezullah Shaikh, FCPS (Gastroenterology). Assistant Professor of Gastroenterology, National Institute of liver & GI Diseases (NILGID), Dow University Hospital, Karachi, Pakistan; 6 Prof. Dr. Nasir Khokhar, MD, FACG. Professor of Medicine, Shifa International Hospital and Shifa Tameer e Millat University, Islamabad, Pakistan

**Keywords:** Sofosbuvir, HCV infection, Rapid virologic response (RVR)

## Abstract

**Objective:**

The uridine nucleotide analogue sofosbuvir is a selective inhibitor of hepatitis C virus (HCV) NS5B polymerase approved for the treatment of chronic HCV infection with genotypes 1 – 4. The objective of the study was to evaluate the interim results of efficacy and safety of regimens containing Sofosbuvir (Zoval) among Pakistani population with the rapid virologic response (RVR2/4 weeks) with HCV infections.

**Methods:**

This is a multicenter open label prospective observational study. Patients suffering from chronic Hepatitis C infection received Sofosbuvir (Zoval) 400 mg plus ribavirin (with or without peg interferon) for 12/24 weeks. The interim results of this study were rapid virological response on week 4. Data was analyzed using SPSS version 21 for descriptive statistics.

**Results:**

A total of 573 patients with HCV infection were included in the study. The mean age of patients was 46.07 ± 11.41 years. Out of 573 patients 535 (93.3%) were treatment naive, 26 (4.5%) were relapser, 7 (1.2%) were non-responders and 5 (1.0%) were partial responders. A rapid virologic response was reported in 563(98.2%) of patients with HCV infection after four weeks of treatment. The treatment was generally well tolerated.

**Conclusion:**

Sofosbuvir (Zoval) is effective and well tolerated in combination with ribavirin in HCV infected patients.

## INTRODUCTION

Hepatitis C virus (HCV) infection is a global health problem, which leads to various complications such as cirrhosis, decompensated liver disease and liver cancer. An estimated 130–150 million people are chronically infected worldwide and 350,000–500,000 HCV-related deaths are reported annually.^1^

HCV infection is responsible for increasing social, economic and health burden worldwide.^2^,^3^ Although the prevalence of HCV infection seems to have declined in the United States^4^,^5^, Western and Northern Europe^6^,^7^, the burden of this disease is escalating in many developing countries like Pakistan.^2^ Lack of awareness, inadequate blood screening facilities, nosocomial transmission and a lack of effective treatments (due to various reasons) are some of the contributing factors for this endemic disease in many developing countries.^3^,^8^-^10^

Based on sequence homology six major HCV genotypes and numerous distinct subtypes have so far been identified.^11^ The distribution of HCV genotypes is highly variable. HCV genotype 3 is endemic in Indian subcontinent.^2^ Multiple studies have identified subtype 3a as the most prevalent HCV variant in Pakistan.^12^,^13^

Treatment for HCV genotype has evolved from pegylated interferon (peginterferon) and ribavirin to include direct-acting antiviral agents. Treatment with interferon is associated with troublesome side effects, including influenza-like symptoms, depression, fatigue, and cytopenias^14^ and requires weekly subcutaneous injections. A substantial proportion of patients with HCV infection are either unable or unwilling to receive an interferon based regimen.^15^,^16^ Therefore, the development of an interferon-free, all-oral treatment regimen would represent an important advancement. Sofosbuvir is a once-daily direct-acting nucleotide polymerase inhibitor that is approved as an oral drug for the treatment of chronic HCV infection.^17^ It is phosphorylated within the host hepatocyte to the active nucleoside triphosphate, which competes with the natural nucleotides, thereby causing termination of RNA replication in the nascent viral genome. The active triphosphate of nucleotide analogues such as Sofosbuvir targets the highly conserved active site of the HCV-specific NS5B polymerase, acting as a non-obligate chain terminator, an effect that is independent of the viral genotype.^18^,^19^

There is lack of published national studies about the use of Sofosbuvir in our population. We are presenting here the early results of efficacy and safety of 12/24 weeks of therapy with regimens containing sofosbuvir in patients with HCV infection. This is one of the first open label large scale clinical study of sobosbuvir (Zoval) under routine clinical practice reporting the initial response on viral load of HCV.

## METHODS

This is a multicenter observational study conducted in various secondary and tertiary care hospitals of Pakistan. Before the start of study, it was registered with ClinicalTrials.gov (NCT02804386). Patient were inducted from the out patients department (OPD) after taking informed consent and fulfilling the inclusion criteria. Patients were assigned registration number according to site code and subject number. Screening assessments included standard clinical, laboratory testing, measurement of serum HCV RNA levels, and HCV genotyping.

Assessments during treatment included measurement of vital signs, physical examinations, electrocardiography, standard laboratory tests and measurement of serum HCV RNA levels.

Sofosbuvir and ribavirin (double regimen) or peg interferon (triple regimen) was prescribed according to international guidelines.^20^ It was solely the physician practice and advice to which patient received double or triple regimen. Sofosbuvir was administered orally at a dose of 400 mg once daily along with ribavirin, which was administered orally as a divided dose according to body weight (1000 mg daily in patients with a body weight of <75 kg and 1200 mg daily in patients with a body weight of ≥75 kg). Peginterferon alfa-2a was administered subcutaneously once weekly at a dose of 180μg if required. Patients results of qualitative/quantitative HCV RNA at week 2/4 were included in this study. Patients were advised to come for follow up at week 4 with HCV results. Adverse events (AE) were inquired on week four during the therapy.

The primary efficacy end point of this study was a rapid virologic response (RVR), which was defined as an HCV RNA level below the lower limit of quantification, (LLOQ; i.e., < 25 IU/mL) at 4 weeks of treatment.

This study was approved by the institutional review board and was conducted in compliance with the Declaration of Helsinki, Good Clinical Practice guidelines, and local regulatory requirements.

Data was entered and analyzed using Statistical Package for Social Sciences SPSS version 21.0.

## RESULTS

A total of 573 patients 290 (56.0%) male and 283 (49.3%) female with HCV infection were included in the study. The mean age of patients was 46.07 ± 11.41 years. Out of 573 patients, 535 (93.3%) were treatment naïve, 26 (4.5%) were Relapser, 7(1.2%) were Non-Responders and 5 (1.0%) were partial responders as shown in Table-I.

**Table-I:** Baseline demographic and clinical characteristic of patients (n=573).

Characteristic	n (%)
***Age (years)***	
Mean ± SD	46.0 ± 11.6
Range	19-80
***Gender***	
Male	290 (50.6)
Female	283 (49.3)
***Body Mass Index***	
Mean ± SD	25.8 ± 5.1
Range	13-44
HCV genotype (197)*	
1	5 (2.5)
2	0 (0)
3	188 (95.4)
4	4 (2.0)
***Previous Treatment Status***	
Treatment naive	535 (93.3)
Relapse	26 (4.5)
Non responder	7 (1.2)
Partial responders	5 (1.0)

* Genotyping and Quantitative RNA levels not done for all patients.

Patient with double regimen (Sofosbuvir, Ribavirin) were 467 (86.7%) however the patient with triple regimen (Sofosbuvir, Ribavirin, Peg-Interferon) were 62 (10.8%). Rapid virological response (RVR), with two weeks of treatment was observed in 84.2% patients and by week four of treatment, 563 out of 573 (98.2%) patients (Table-II). Nineteen out of 23 (82.6%) cirrhotic patients who received triple therapy, responded while the response rate was 88.4% and 85.7% in relapsers and non-responders to interferon therapy respectively.

**Table-II:** Rapid Virologic Response during the Treatment with Sofosbuvir (Zoval).

Response*	n (%)
***All patients during treatment***	
Week 2	16/19 (84.2)
Week 4	563/573 (98.2)
Cirrhotic patients (triple therapy)	19/23 (82.6)
Relapse patients to interferon	23/26 (88.4)
Non responder to interferon (triple therapy)	6/7 (85.7)
***With or without interferon***	
Double therapy	497 (86.7)
Triple Therapy	76 (13.2)

* An HCV RNA level of 25 IU per milliliter (lower limit of quantification).

The treatment was generally well tolerated and side effects were mild to moderate and included fatigue 136 (23.7%), headache 54 (9.4%), anemia 49 (8.5%) and nausea 6 (1.0%). Only two patients discontinued treatment due to adverse effects. There was one reported death among patients included in this study. This patient had a co infection with hepatitis E virus and died due to acute liver failure. (Table-III).

**Table-III:** Treatment discontinuation and adverse events reported during the study period.

Variable	n (%)
Discontinuation of treatment due to adverse event	2 (0.34)
*Adverse Events*	
Fatigue	136 (23.7)
Nausea	6 (1.0)
Headache	54 (9.4)
Insomnia	1 (0.1)
Pruritus	3 (0.52)
Urticaria	1(0.1)
Anemia	49 (8.5)
Cough	2 (0.3)
Arthralgia	1 (0.1)

## DISCUSSION

In this open-label, single-group study of sofosbuvir in patients with HCV infection, more than 98% of patients receiving treatment had a rapid virologic response (RVR) at four weeks which is comparable to other published studies.^21^ As this is an ongoing study therefore SVR at week 12 will be reported later. Our four weeks treatment response is in agreement with other controlled studies which reported results from 97-99% at week four of treatment.^21^ Taking account of under general practice conditions the results of the study are very good. In phase two trials, a regimen of 400 mg of sofosbuvir plus peginterferon– ribavirin for 12 or 24 weeks resulted in rates of sustained virologic response of 87 to 92% in previously untreated patients with HCV Genotype-1 infection.^22^,^23^ Patients with genotype 4 or 6 infection also had high rates of sustained virologic response with a 24-week regimen of sofosbuvir plus peginterferon– ribavirin.^22^ In another phase two trial, all 40 previously untreated patients with HCV Genotype- 2 or 3 infection had a sustained virologic response with 12 weeks of treatment with sofosbuvir plus ribavirin (with or without peginterferon).^24^ In another double-blind study with 64 patients who were taking one of three once-daily doses of oral sofosbuvir with the strength of 100, 200, or 400 mg) or placebo plus PegIFN/RBV for 28 days. It revealed that on 28 day the response rate of Sofosbuvir 200 and 400 showed optimal results.^25^

In our study 19 out of 23 cirrhotic patients (82.6%) receiving sofosbuvir responded. Response rate in cirrhotic patients varies in different studies. In a phase 3 trial in patients infected with HCV genotype 1, (FISSION study) treatment-naive patients, including a subgroup with compensated cirrhosis, 89% achieved SVR after 12 weeks of treatment with sofosbuvir and peginterferon or ribavirin.^26^ In Genotype-3 the response rates were lower as compared to Genotype 2 (56% vs. 97%). The response rate was lower in cirrhosis patients as compared to non-cirrhosis in genotype 1. 25

Fatigue, headache, and nausea were the most frequent adverse effects (AEs) as reported in other studies of sofosbuvir in ribavirin-containing regimens.^24^ AEs were mild to moderate, clinically manageable, and less frequent than are seen with interferon-containing regimens and none of the patients discontinued treatment due to side effects. There was one reported death during treatment which was due to acute liver failure as patient was diagnosed to have co-infection with Hepatitis E virus. Overall, the combination of sofosbuvir and ribavirin was effective and well tolerated by previously non-responders and treatment-naive patients with chronic HCV infections.

### Limitations of the study

As this is an observational study in real life clinical practice so genotyping and Quantitative RNA levels were not done in all patients though it is very important for HCV treatment analyses and helpful for proper treatment planning. SVR12 is the main efficacy parameter to evaluate success of any treatment of HCV, but in this study we are only reporting interim analysis of EVR2 and EVR4.

To the best of our knowledge this is perhaps the first reported experience of Sofosbuvir in our Pakistani population and will help in building confidence regarding use of sofosbuvir (Zoval) in clinical practice. Hopefully this regimen will address the rising clinical need for effective oral interferon-free treatment options in patients with HCV infections and both to treatment naïve and those who have previously not responded to peg interferon.

## CONCLUSION

Sofosbuvir (Zoval) with ribavirin is effective and well tolerated with and without interferon in patients with HCV infection among Pakistani population.

## Authors’s Contibution

**ZA** with Sponsor conceived the idea, designed the study, helped in CRF development and data collection, oversee data anaylsis.

**ZA, MS, MJ, MAS** helped in data collection and patients suprevision.

**ZA, MS, MJ, MAS HS, NK** approval of the final version to be published.
